# Supplementary material for: Crawling and Gliding: A Computational Model for Shape-Driven Cell Migration
Source: PLoS Comput Biol. 2015 Oct 21;11(10):e1004280. doi: 10.1371/journal.pcbi.1004280 (PMC4619082; doi:10.1371/journal.pcbi.1004280)
Supplement: S1 Code — (ZIP) [file pcbi.1004280.s012.zip › release/tst/doc/html/parameter_8cpp.html]

Tissue Simulation Toolkit: parameter.cpp File Reference


Tissue Simulation Toolkit
 0.1.4.1


- Main Page
- Namespaces
- Classes
- Files

- File List
- File Members

Functions |
Variables

parameter.cpp File Reference

`#include "parameter.h"`  
`#include <cstdio>`  
`#include <cstring>`  
`#include <cstdlib>`  
`#include <cerrno>`  
`#include <iostream>`  
`#include "output.h"`  
`#include "parse.h"`

Include dependency graph for parameter.cpp:

|  |  |
| --- | --- |
| Functions | |
| const char \* | sbool (const bool &p) |
|  | |
| ostream & | operator<< (ostream &os, Parameter &p) |
|  | |

|  |  |
| --- | --- |
| Variables | |
| Parameter | par |
|  | |

## Function Documentation

|  |  |  |  |
| --- | --- | --- | --- |
| ostream& operator<< | ( | ostream & | *os*, |
|  |  | Parameter & | *p* |
|  | ) |  |  |

References Parameter::Write().

|  |  |  |  |  |  |
| --- | --- | --- | --- | --- | --- |
| const char\* sbool | ( | const bool & | *p* | ) |  |

Referenced by Parameter::Write().

## Variable Documentation

Parameter par

Referenced by Info::Menu(), and QtGraphics::TimeStepWrap().


---

Generated on Thu Aug 14 2014 22:04:01 for Tissue Simulation Toolkit by  

 1.8.6
